# Etodolac Single Dose Metabolic Profile Elucidation: Pharmacokinetics and Adverse Events in Healthy Volunteers

**DOI:** 10.3390/ph18010082

**Published:** 2025-01-11

**Authors:** Karen Sánchez-Luquez, Anne Michelli Reis Silveira, Salvador Sánchez-Vinces, Alex Ap. Rosini Silva, Joyce Barreto, Rhubia Bethania Socorro Lemos de Brito, Caroline de Moura Garcia, Ana Lais Vieira, Marcia Ap. Antonio, Patrícia de Oliveira Carvalho

**Affiliations:** 1 Health Sciences Postgraduate Program, São Francisco University—USF, Bragança Paulista 12916-900, SP, Brazil; ksanchezluquez@gmail.com (K.S.-L.); anne.silveira@unifag.com.br (A.M.R.S.); salvdor.vinces@mail.usf.edu.br (S.S.-V.); alexrosinisilva@hotmail.com (A.A.R.S.); 2 Integrated Unit of Pharmacology and Gastroenterology (UNIFAG), São Francisco University—USF, Bragança Paulista 12916-900, SP, Brazil; joyce.barreto@unifag.com.br (J.B.); marcia.antonio@unifag.com.br (M.A.A.); 3 Althaia S.A. Indústria Farmacêutica, Atibaia 12952-820, SP, Brazil; rhubia.brito@althaia.com.br (R.B.S.L.d.B.); garcia.carol100@gmail.com (C.d.M.G.); analais.vieira@althaia.com.br (A.L.V.)

**Keywords:** etodolac, metabolomics profile, metabolic pathways, pharmacokinetics

## Abstract

Background/Objectives: This study investigates the metabolic profile of a single dose of etodolac in healthy volunteers, focusing on pharmacokinetics, clinical parameters, and metabolomic variations to identify biomarkers and pathways linked to drug response, efficacy, and safety. Methods: Thirty-seven healthy volunteers, enrolled after rigorous health assessments, received a single dose of etodolac (Flancox^®^ 500 mg). Pharmacokinetic profiles were determined using tandem mass spectrometry analysis, and the metabolomic profiling was conducted using baseline samples (pre-dose) and samples at maximum drug concentration (post-dose) via liquid chromatography coupled with a quadrupole time-of-flight mass spectrometer. Network analysis was employed to interpret the data. Results: Correlations were observed between metabolomic profiles and pharmacokinetic parameters as well as clinical characteristics. Notably, metabolites derived from arachidonic acid, such as prostaglandins and leukotrienes, were linked to etodolac’s pharmacokinetics. Other metabolites involved in pathways like cholesterol biosynthesis, bile salts, riboflavin, and retinoic acid signaling were correlated with hematological and liver function parameters. These findings are consistent with the infrequent adverse events reported by participants, including hematological and biochemical changes in liver function. Conclusions: A set of metabolites was identified in possible associations between specific pathways and unusual side effects, comparing the metabolic profiles before and after doses of etodolac. Our results highlight the importance of optimizing drug therapy and minimizing adverse events by taking into account individual metabolic profile information.

## 1. Introduction

The World Health Organization (WHO) recommends using non-steroidal anti-inflammatory drugs (NSAIDs) to treat mild, moderate, and severe inflammation/pain [1]. Etodolac (ETO) is the gold standard for osteoarthritis management and does not inhibit gastric prostaglandins [2]. In Brazil, ETO, a NSAID, remains of clinical and commercial interest. The website of the Brazilian regulatory authority [3] reports that bioequivalence studies of new formulations are currently underway. Therefore, adequate knowledge of the effects of drugs used in inflammation and pain management, from physiology to pharmacology, is essential for appropriate and optimized inflammation/pain management [4].

ETO is a NSAID classified by the Biopharmaceutics Classification System as BCS class II (dissolution rate-dependent absorption). ETO is highly safe and effective in relieving pain and controlling inflammation. ETO has poor solubility and a dissolution rate that could result in variable oral absorption and inconsistent analgesic responses [5]. NSAIDs inhibit the synthesis of prostaglandin (PG) by cyclooxygenase (COX), an enzyme of which two isoforms (COX-1 and COX-2) with different functions have recently been identified. Selective cyclooxygenase-2 (COX-2) inhibitors differ from traditional NSAIDs in their preferential inhibition of the COX-2 receptor over the COX-1 receptor. COX1 is essential for the maintenance of gastrointestinal and renal function, while COX-2 converts arachidonic acid into prostaglandins. Prostaglandins are potent mediators of inflammation, pain, and fever. ETO exerts its therapeutic effects primarily by inhibiting the enzyme cyclooxygenase 2 (COX-2) [5,6,7,8]. So far, the results obtained in different clinical trials indicate that these selective COX-2 inhibitors are as effective in combating inflammation and hyperalgesia as conventional NSAIDs, with the added benefit of producing fewer gastrointestinal lesions [9]. In this context, it is also interesting to evaluate the impact of selective COX2 inhibitors and check their possible influences from a metabolic approach.

Pharmacokinetics, the ability to predict the concentration of a drug in the body over time, including absorption, distribution, metabolism, and excretion—ADME—is a traditional method of predicting pharmacokinetics based on a drug’s chemical structure [10]. However, these methods have limitations. They are not always able to predict the behavior of a drug in a given individual. Pharmacometabolomics offers several advantages in drug development by integrating metabolomics with pharmacokinetics. Analysis of the metabolome improves the accuracy and sensitivity of the identification of factors that influence drug absorption, distribution, and/or biotransformation, as well as potential safety events [11]. It is often used with other drugs, and it is important to know its metabolomics in single doses [10]. Prolonged or high-dose use of ETO is associated with an increased risk of peptic ulcer and gastrointestinal bleeding; in addition, ETO may affect renal function, particularly in patients with pre-existing risk factors, and may increase the risk of cardiovascular events such as myocardial infarction and stroke [12], although the exact pathways by which this occurs remain to be elucidated. Song et al., 2017, have primarily focused on traditional pharmacokinetic (PK) parameters and bioequivalence assessments. While these studies provide valuable insights into drug ADME, they have not incorporated a metabolomic approach [13].

This study aims to investigate the relationship between variations in the metabolic profile and the pharmacokinetics as well as laboratorial and clinical effects of a single dose of etodolac in healthy subjects. It seeks to evaluate the potential of metabolomics to identify specific pathways and metabolites of drug response in the assessment of drug efficacy and safety. To predict key pharmacokinetic parameters and potential safety issues, pharmacometabolomics strategies using ultra-performance liquid chromatography-quadrupole time-of-flight mass spectrometry (UPLC-QToF) were applied to study healthy human plasma samples. The results of this study may help to better elucidate the involved metabolites and possible biological mechanisms underlying the use of a single dose of etodolac through metabolomic approaches, thus advancing personalized strategies.

## 2. Results

### 2.1. Volunteers’ Clinical and Laboratory Characteristics

**Figure 1:**
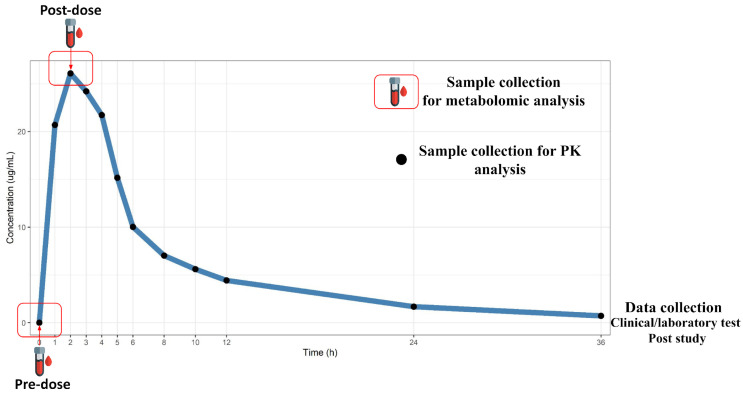
Representative diagram of sample/data collection points used in this study.

The study was completed with 37 healthy adult participants, 18 female and 19 male, aged between 18 and 41 years old (mean 28.03 ± SD 6.8) and with a body mass index between 18.7 and. 29.1 kg/m^2^ (mean 24.2 ± SD 2.7). Details of the experiment are shown in Figure 1 and methods, as well as in Table S1. In this study, there were 19 adverse events recorded -that is, individuals with results outside the reference values used to define individuals as healthy at baseline- (in its entirety of predictability: not expected and intensity: mild), which are presented in the Summary of Adverse Events in Table S2.

### 2.2. Determination of Etodolac Pharmacokinetics

**Figure 2:**
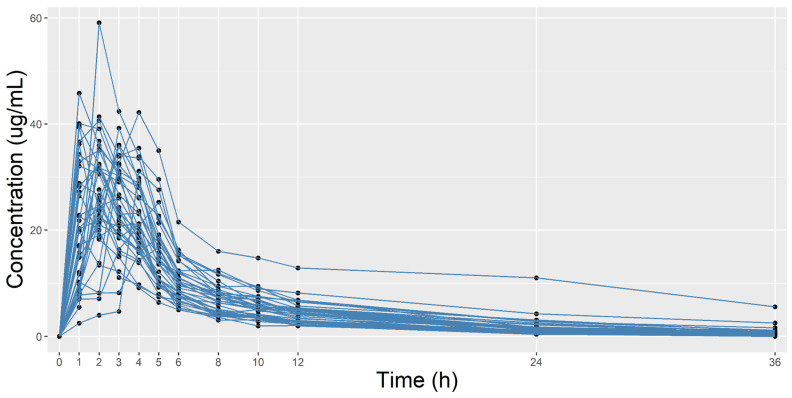
Plot of all individual pharmacokinetic curves.

Figure 2 plots individual pharmacokinetics curves (concentration vs. time). In brief, the overall pharmacokinetic parameters were as follows: area under the curve from time zero to time t (AUC0-t) (h·μg/mL) = 202.83 (72.05), maximum concentration (Cmax) (μg/mL) = 35.54 (9.14), time to maximum concentration (T_max_) (h) = 2.24 (1.11), elimination rate constant (Kel) (1/h) = 0.09 (0.02), and half-life (T1/2) (h) = 8.2 (2.73). Table S3 shows the pharmacokinetics parameters for each participant.

### 2.3. Metabolomic Analysis

**Figure 3:**
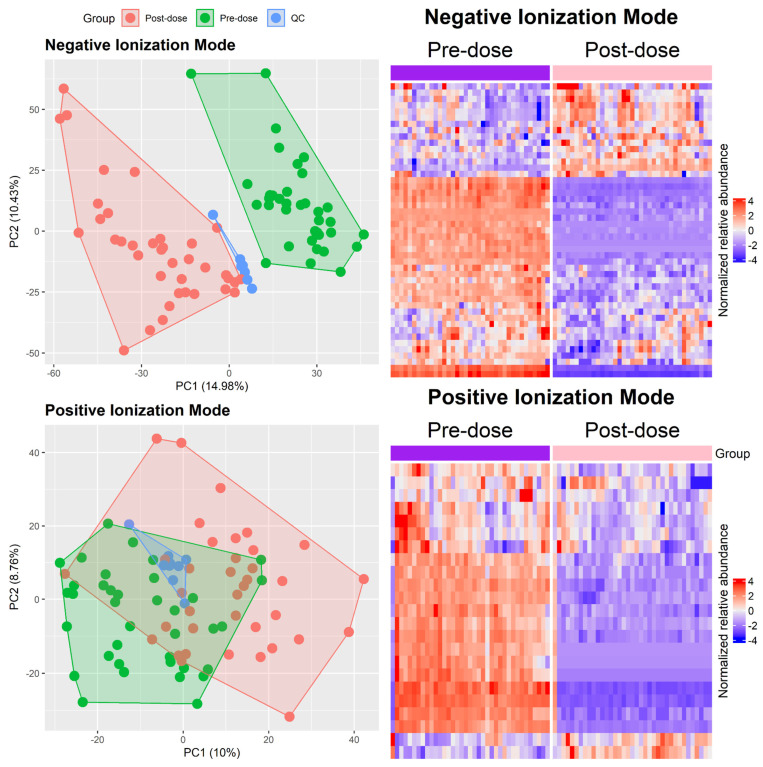
PCA plots for negative and positive ionization modes and normalized relative abundance heatmaps for selected compounds, detected in negative (**left**) and positive (**right**) ionization modes.

The untargeted analysis detected 2466 features in the positive mode and 4777 in the negative mode. Our feature selection model detected 1390 features in negative ion mode and 290 in positive ion mode as differences at the pre- and post-dose time points. Of these 1680 differential features, 70 were putatively identified as metabolites (Table S4). Figure 3 shows the dispersion of participants and QC samples using principal component analysis and the heat maps for the relative abundance of selected and identified features.

### 2.4. Enrichment Analysis

The pathway enrichment analysis (Table S4) used the 70 selected and putatively identified features as input. With an adjusted p-value by FDR ≤ 0.05, there were 131 pathways. Table S5 shows the results generated by the RaMP package. The Reactome database was used for the highly related pathways in the results. Some Wikipath pathways are their own versions of the Reactome pathways. In this way, the most important functional or structural features between the enriched pathways can be understood and discussed, including the following: digestion and absorption = R-HSA-8963743 (FDR *p*-value = 0.0011); digestion of dietary lipid = R-HSA-192456 (FDR *p*-value = 0.00015); transport of vitamins, nucleosides, and related molecules=R-HSA-425397 (FDR *p*-value = 0.00015); synthesis of bile acids and bile salts = R-HSA-192105 (FDR *p*-value = 0.026); alpha-linolenic acid metabolism = map00592 (FDR *p*-value = 0.026); metabolism of alpha-linolenic acid (WP4586) (FDR *p*-value = 0.042); and prostaglandin and leukotriene metabolism in senescence (WP5122) (FDR *p*-value = 0.044).

### 2.5. Community Network 

After module discovery using community network analysis, 12 modules representing sets of metabolites associated with PK parameters and clinical/laboratory participant characteristics (Table S6). The pre-dose metabolome dataset formed five modules with 19 edges, where 8 metabolites were significantly associated with systolic pressure, 9 characteristics in serum, and 1 in urine-related. In the post-dose metabolome dataset, 35 metabolites were significantly associated with 5 PK parameters, and 13 characteristics in serum by 67 edges that formed seven modules.

**Figure 4:**
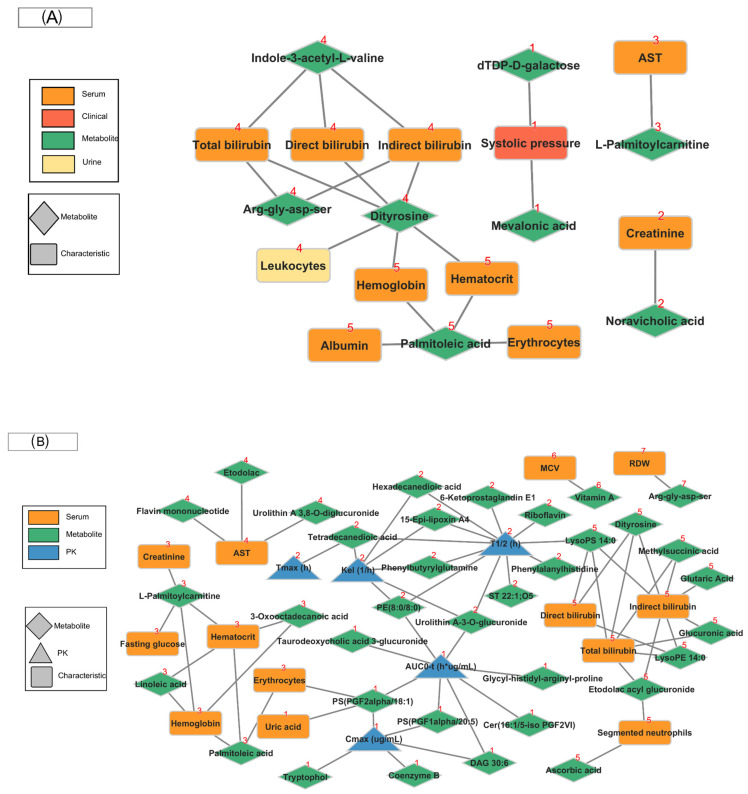
Community networks were constructed using PK parameters, clinical and laboratory traits, and identified metabolites at (**A**) pre-dose and (**B**) post-dose. A red label above each node indicates the number of communities or modules.

Figure 4A shows the community networks in the pre-dose period, and Figure 4B shows the community network in the post-dose period. Table S6 shows the complete lists of members for the pre-dose and post-dose communities.

### 2.6. Integrating Pathway and Network Results

The set of metabolites associated with the PK parameters and/or laboratorial and clinical characteristics in each module derived from the network analysis was integrated, according to possible functional/biological pathway correspondence, with the results obtained in the enrichment pathway derived from the differential metabolites between the pre and post-dose time points, as shown in Table 1. These combined results showed the association between metabolism at pre-dose with systolic blood pressure and laboratory tests such as renal function. Correlations were found between the relative abundance of certain metabolites after ETO administration with ADME parameters, laboratory-related liver and kidney function, and white blood cell counts.

## 3. Discussion

We used metabolite set enrichment and community network analysis to detect and understand the associations of differential metabolic profiles with pharmacokinetics, clinical, and laboratory parameters in healthy volunteers before and after receiving a single dose of ETO. Significant associations were found between the pre and post-dose ETO metabolomic profiles.

The baseline demographics did not show any significant differences in terms of gender, age, and body mass index. Infrequent but mild adverse events were reported, as discussed below. The values of the pharmacokinetic parameters obtained in this study are comparable to those reported so far in the literature for ETO [14,15].

### 3.1. Mapping Predictive Associations

In this context, metabolic changes observed in individuals after a single dose of ETO may result from primary activity or secondary physiological disturbance. The metabolism of prostaglandin and leukotriene metabolism were biological pathways that represented significant changes in our study. Our results support previous reports by previous studies that NSAID drugs potentiate the release of anaphylactic slow-reactants, which are now known to be a mixture of the leukotrienes C4, D4, and E4 [6,15].

The association of metabolite sets at pre-dose with pharmacokinetic parameters may explain or even predict biological pathways related to the drug’s ADME. Although the pathways obtained in the enrichment analyses do not explain the module network functional activity, they do explain functionally where the metabolite associated with the module may act.

#### 3.1.1. Association with Pharmacokinetic Parameters

At pre-dose, we found no association between metabolite networks and pharmacokinetic parameters. That is, none of the differential metabolites between pre-dose and post-dose were able to ’predict’ parameters related to the pharmacokinetics of ETO in this study.

#### 3.1.2. Laboratory/Clinical Parameters

The baseline metabolomic assessment shows metabolites mainly involved in cholesterol biosynthetic pathways, cholesterol synthesis disorders, and omega-9 fatty acid synthesis are correlated with clinical (systolic blood pressure) and laboratory signs plasma blood tests (total cholesterol level, renal function, biliary pigment, and liver function) and urine with white blood cell count during the post-trial evaluation of participants.

The production of mevalonic acid by the enzyme 3-hydroxy-3-methylglutaryl-coenzyme A (HMG-CoA) reductase is the rate-limiting step in the biosynthesis of cholesterol. Also, the dTDP-D-galactose belongs to the class of organic compounds known as cholesterols and derivatives. Cholesterol fulfills a number of biological functions and is necessary for the successful maintenance of cellular homeostasis. It plays a central role in maintaining the rigidity and fluidity of cell membranes, acts as a precursor to bile acids, and aids in the synthesis of steroids and vitamin D [16]. Aldosterone is synthesized in the adrenal cortex. It acts mainly on the renal tubules, stimulating potassium excretion and the uptake of sodium and water. The ultimate effect is an increase in blood pressure [17]. Cholesterol also serves as a precursor for bile acids and influences blood pressure through the synthesis of aldosterone.

Omega-3, -6, and -9 fatty acids are unsaturated fatty acids that have multiple biological effects and health benefits. Different omega-9 fatty acids have different pharmacological effects. These include modulation of inflammation, lipid, cardiovascular, and cancer disorders [18]. Palmitoleic acid was identified as a lipokine that has important positive effects on metabolic disorders. [19]. In our study, this metabolite was associated with albumin and hemoglobin levels at T0, which may be useful/important in assessing the nutritional status of the individual and identifying possible renal or hepatic problems prior to ETO administration.

Total, direct, and indirect bilirubin and urinary leukocytes are biomarkers that can provide a more complete picture of an individual’s health status, particularly in the context of liver, inflammatory, and oxidative stress-related diseases. Recent studies suggest that human serum albumin, the major plasma protein, may have a direct vasculoprotective antioxidant effect [20]. Dityrosine is a product of radical oxidation [21]. Previous studies have shown that dityrosine production is approximately twofold increased in uremic human serum albumin from dialysis patients. Dityrosine may help phagocytes respond to inflammation [20]. The process described here may also be associated with the enrichment of several oxidative stress-related pathways, such as the cellular response to hypoxia and the effects of nitric oxide, as shown in Table S5.

### 3.2. Exploring Explanatory Parameters

The results indicate a possible trend toward a cause and/or effect relationship of ETO in the body. In fact, correlations were obtained between certain metabolites after ETO administration and pharmacokinetic and/or clinical parameters.

#### 3.2.1. Pharmacokinetic Parameters

We found a correlation between AUC0-t/Cmax and PS(PGF1α/20:5) and PS(PGF2α/18:1) in our findings. The anionic phospholipids, phosphatidylserine (PS), and phosphatidylglycerol (PG) are naturally occurring molecules with anti-inflammatory properties. PS, a crucial component of cell membranes, undergoes oxidation [22]. This oxidation endows PS with new properties that can affect various biological processes. These oxidized forms of PS may play a role in regulating the inflammatory response and immunomodulatory activity [22]. Additionally, they have been linked to increased activity of certain drug transporters [23].

In addition, tryptophol was identified, showing a more than fourfold increase between T0 and Tmax, and correlating positively with Cmax. The relationship between tryptophol metabolism and etodolac pharmacokinetics may be indirect and complex. Tryptophol, a catabolite of tryptophan, participates in multiple metabolic pathways that can influence liver enzyme activity, such as tryptophan metabolism [24,25]. These enzymes are responsible for the biotransformation of etodolac. However, further research is needed to support this hypothesis.

About the Kel, we obtained evidence for two metabolites associated with arachidonic acid metabolism, specifically the 15-epi-lipoxin A4 [26]. This metabolite increases in relative abundance at Tmax compared to T0. It is positively associated with the elimination constant and negatively associated with T1/2. The 6-ketoprostaglandin E1 [27] increases twist time in relative abundance at Tmax compared to T0 and has been shown to be negatively associated with T1/2. Our results tend to support direct or indirect disturbances after ETO use [6,28].

#### 3.2.2. Laboratory/Clinical Characteristics

Our results on clinical traits networks indicate variation in plasma concentrations of metabolites involved in fatty acid metabolism. They are either substrates, products, or regulators. Directly involved in the energy-producing pathway are L-palmitoylcarnitine and 3-oxooctadecanoic acid. Linoleic acid and palmitoleic acid are involved in several physiological processes. These include inflammation and insulin sensitivity. Alterations in their metabolism can affect cellular integrity, renal function (creatinine), and glycemic regulation (glucose). In this network, the metabolite palmitoylcarnitine decreased in abundance in the blood after ETO administration, correlating with hematocrit values. This metabolite may be involved in pathways associated with retinoic acid signaling, which is implicated in several cellular processes, including response in vascular endothelial cells, and could influence hematological parameters [29].

Etodolac abundance showed a negative correlation with serum oxaloacetic transaminase (TGO). ETO, like other NSAIDs, can affect hepatic metabolism, which may be reflected in changes in the levels of enzymes such as TGO [15]. Although they are generally well tolerated by the liver, prolonged use or high doses may damage liver cells. Consequently, flavin mononucleotide increased relative abundance post-ETO dose and correlated negatively with serum TGO. Consistent with this finding, we also found a correlation between etodolac acyl glucuronide (an ETO metabolite) and serum bilirubin levels.

Variation plasmatic of flavin mononucleotide was found. Riboflavin is known to be involved in a variety of redox reactions central to human metabolism through the cofactors flavin mononucleotide (FMN) and flavin adenine dinucleotide (FAD) [30]. Insufficient riboflavin intake would, therefore, be expected to lead to disruptions in intermediary metabolic steps, with functional implications [31]. A recent publication investigating the impact of a biliary injury on the recurrence of biliary cancer and benign disease after liver transplantation analyzed possible underlying mechanisms associated with FMN [32]. Therefore, it may be essential to consider both the direct effect of the drug and the patient’s nutritional status when evaluating liver test results during treatment with etodolac.

Our study also showed a pre/post increase in vitamin A, considered an important micronutrient in the mammalian diet, which exists in three forms: retinal, retinol, and retinoic acid, the latter being the most metabolically active [33], retinoic acid, which acts through nuclear retinoic acid receptors and is a potent regulator of pattern formation during embryonic development, which is required for the homeostasis of adult tissues [34].

#### 3.2.3. Adverse Event-Related Association

Prolonged or high-dose use of ETO is associated with an increased risk of peptic ulcer, gastrointestinal bleeding, renal dysfunction, myocardial infarction, and stroke [12]. Our unique dose study of ETO showed an infrequent adverse events profile that included changes in hematological profile and biochemical liver function. However, our results are limited in correlating these findings with possible associations between the observed adverse events and the properties of ETO as a selective COX-2 inhibitor. Our module and enrichment analysis consistently shows associations between metabolite sets and hemogram and liver function at both pre and post-dose. Network analysis showed consistent pre- and post-dose ETO associations between specific fatty acids (palmitoleic acid, linoleic acid) and erythrocyte levels, which may indicate an effect on membrane phospholipid synthesis and cell survival.

In addition, consistent pre- and post-dose ETO associations were observed between arg-gly-asp-ser, L-palmitoylcarnitine, dityrosine (Table S6), and biochemical liver function. It is plausible to think of this association since the liver plays a central role in the metabolism of amino acids and is responsible for their synthesis, degradation, and interconversion. Changes in the levels of these compounds may reflect liver dysfunction. Other associations were observed, although the underlying mechanisms remain unclear. To fully understand these associations and their clinical implications, further research is needed to evaluate changes in the metabolic profile after long-term use of ETO.

### 3.3. Limitations and Conclusions

Several study limitations should be considered, such as the sample size, which limits the power of the study to detect small effects. The genetic conditions of the participants are important factors that may influence the pharmacokinetics of etodolac, such as variability in its metabolism, classically through genetic polymorphisms in UGT1A9 and CYP2C9 [35], were not accessible, so their impact on drug metabolism was not evaluated. Unidentified compounds could also confound the results. However, the controlled trial design of bioequivalence studies minimizes confounding factors, and the untargeted metabolomic analysis offers a comprehensive view of metabolic changes in patients, with identification accuracy deemed acceptable based on established criteria.

The complex interaction between ETO and the metabolic landscape of the human body is highlighted in this study. Pathways involved in the inflammatory process seem to be affected following ETO administration. Variations observed pre- and post-dose ETO dosing may explain rare adverse events. Our findings reinforce the significant contribution of a metabolomic approach to a comprehensive understanding of PK profiles. By providing a predictive, sensitive, and specific assessment of drug response and adverse effects, our results demonstrate the potential of metabolomics as a valuable tool for the identification of specific metabolites and pathways in the understanding of individual variability in response to a drug in clinical trials. The identification of specific pathways and metabolites associated with adverse events in healthy volunteers provides the basis for the development of predictive biomarkers in patient populations, for example, in the context of inflammation and/or pain. This could enable clinicians to proactively monitor patients for potential side effects and adjust treatment accordingly. For future perspectives, we suggest studies incorporating metabolomic analyses in studies of prolonged use or high doses of ETO to validate our initial findings. We also suggest the initiation of studies investigating the metabolites set listed in our study as possible relevant molecules for more specific biological tests related to the mechanism of action of ETO to investigate the clinical utility of these findings in patient populations and explore the potential for metabolomic profiling-based personalized medicine. We also suggest considering genetic factors that may influence these pharmacophenotypic relationships.

## 4. Materials and Methods

### 4.1. Study Design and Healthy Human Subjects

This study was a nested, single-dose, open-label, randomized pharmaco-bioequivalence study conducted under biosafety and ethical standards (Figure 1). The data used in this study were obtained from 37 healthy volunteers administered the reference ETO drug (Flancox® 500 mg, by Apsen Farmaceutica, São Paulo, Brazil) and selected for the study after assessment of their health status by clinical evaluation before and after ETO administration, by physical examination and electrocardiogram, and by a comprehensive set of laboratory tests (systolic pressure, diastolic pressure, heart rate, temperature clinical, total cholesterol seric, triglycerides seric, urea seric, creatinine seric, fasting glucose seric, uric acid seric, aspartate aminotransferase seric, alanine aminotransferase seric, total bilirubin seric, direct bilirubin seric, indirect bilirubin seric, alkaline phosphatase seric, total protein seric, albumin seric, hemoglobin seric, erythrocytes seric, hematocrit seric, mean corpuscular volume (VCM) seric, mean corpuscular hemoglobin (MCH) seric, corpuscular hemoglobin concentration (CHCM) seric, red cell distribution width (RDW) seric, leukocytes seric, segmented neutrophils seric, eosinophils seric, lymphocytes seric, monocytes seric, platelets seric, density urine, leukocytes urine, erythrocytes urine, pH urine), as listed in detail in the Table S1. Venous blood (5 mL) was collected for development and validation analysis using tubes containing ethylenediaminetetraacetic acid (EDTA). After collection, the samples were centrifuged at 2000× *g* for 10 min at 4 °C. Subsequently, the plasma was carefully collected and stored at −80 °C for later analysis. The same parameters were used to monitor adverse events for safety and tolerability. None of the subjects were smokers, alcoholics, or had a history of drug abuse.

### 4.2. Ethics

The protocol complied with Brazilian legislation on human clinical research and was duly approved by the Research Ethics Committee. Institutional Review Board (IRB) approval for the study was obtained under protocol number 58683422.6.0000.5514, dated 1 May 2022. All subjects gave their written informed consent and were free to withdraw from the study at any time.

### 4.3. Determination of Pharmacokinetic Profile

The analytical method used to calculate the pharmacokinetic parameters was validated according to Resolution No. 27/2012 of the Agência Nacional de Vigilância Sanitária (ANVISA). In this study, an LC-MS/MS system was used to quantify ETO. Chromatographic separations were performed using an LC-20AD analytical pump (Shimadzu, Kyoto, Japan) and a SIL-20A HT autosampler (Shimadzu, Kyoto, Japan), coupled to a Quattro Micro (Micromass, Newcastle, UK) system for triple quadrupole mass spectrometry analyses with an electrospray ionization (ESI) source (Waters, Newcastle, UK). The targeted analysis for ETO was detected by a multiple reaction monitoring (MRM) transition of 286 > 242, and mefenamic acid (Internal Standard) was detected by an MRM transition of 240 > 196 in negative acquisition mode. Data were collected using MassLynx 4.1 (Waters, Newcastle, UK). Further information on the validation method can be found in Supplementary Materials and Methods. WinNonLin 8.3 software (Pharsight, New Jersey, USA) was used to calculate pharmacokinetic parameters using non-compartmental statistics.

### 4.4. Metabolomic Profile

Samples were collected at two times: (i) baseline samples (time 0 h), termed the pre-dose group, and (ii) samples at maximum drug concentration (Cmax), termed the post-dose group. Samples were randomized, and 25 μL of each was collected to form a pooled sample for quality control (QC). An aliquot of plasma (200 μL) was extracted by adding cold methanol (400 μL) for protein precipitation. The samples were then vortexed for 30 s and centrifuged at 12,879× *g* for 10 min at 4 °C. Finally, the supernatant (400 μL) was collected and dried over a stream of nitrogen gas (N_2_). Samples were resuspended with 200 µL of acetonitrile (ACN)/water (H_2_O) (1:1 *v*/*v*). Sample extraction and analysis were randomized to reduce instrumental and biological bias. QC samples were analyzed every 10 samples to check for variations in extraction and system stability.

The analysis was adapted [36] and performed on an ACQUITY® FTN H Class (Waters, Newcastle, UK) liquid chromatography (UPLC) coupled to a XEVO-G2XS (QToF) quadrupole time-of-flight mass spectrometer (UPLC-QToF) (Waters, Manchester, UK) equipped with an electrospray ionization (ESI) source operated in the positive and negative ionization mode, separately. An ACQUITY UPLC ® CSH C18 column (2.1 mm × 100 mm × 1.7 µm, Waters, Newcastle, UK) was employed as a stationary phase. The mass spectrometer was operated in MSE mode (DIA, data independent analysis) with an m/z range of 50–1200 Da and an acquisition time of 0.5 s per scan. MSE analysis was operated at 6 eV for low collision energy and a ramp of 20–50 eV for high collision energy. See Supplementary Materials and Methods for more information on metabolomic analysis.

#### 4.4.1. Data Processing and Selection of Features

The LC-MS raw data files were processed using Progenesis™ QI v2.4 software (Non-linear Dynamics, Newcastle, UK), which allows adduct selection, peak alignment, deconvolution, and compound annotation via MSE experiments. Progenesis QI generates an intensity table of features labeled according to their nominal masses and retention times as a function of intensity. The SERRF package [37], a QC-based signal correction method implemented in R 4.2.3 statistical programming language [38], was used to correct any potential signal drift. Zeros were then replaced by half of the smallest positive value in the original dataset. An interquartile range criterion (lowest 10% by IQR rank) was used to filter out features with constant or near-constant values. Features with a relative standard deviation (RSD) in the QC sample data greater than 20% were filtered out. The filtered data were transformed using a logarithmic scale and a Pareto scale.

The limma method [39], implemented in the MetaboAnalystR 3.0 [40] package in R, was used to select features with differential abundance between the pre-dose and post-dose groups. After the empirical Bayes fit and the covariate adjustment applied with the limma method, we selected features with an FDR -≤ 0.05. First, the covariates or confounding variables were selected according to the literature [41] for known characteristics: sex, age, and body mass index (BMI). The bioequivalence period variable was also considered a covariate. The participant tag variable was considered a blocking factor to preserve the relationship between samples before and after etodolac administration.

#### 4.4.2. Identification of Metabolites

Through the low and high energy acquisition facilitated by MSE, we obtain data on precursor ions (low energy) and fragments (high energy) within a single spectrum. The characterization of molecules is based on mass precision (≤5 ppm), fragmentation profile (≤10 ppm), isotopic similarity (>70%), and biological significance. For Progenesis PQI data compatibility, the in-house software v1.0 ‘SDF2PQI’ was employed to improve fragment matching, as it converts files from external SDF-based spectral libraries, allowing their use in Progenesis PQI [42]. MassBank of North America (MoNA) [43], the Human Metabolome Database (HMDB) [44], and the LIPID MAPS structure database [45] were used as libraries.

### 4.5. Enrichment Pathway Analysis

A starting approach to interpreting the metabolite differential set was the knowledge-driven pathway enrichment. The package RaMP 2.0 was used [46], developed in R. This tool queries three different databases to enrich sets: KEGG [47], WikiPathways [48], and Reactome [49]. The RaMP analysis employed the following criteria: a minimum of one metabolite hit per pathway, pathway size ranging from five to 150 metabolites, and FDR value < 0.05. The output comprised a collection of enriched pathways, along with their respective sources, unique identifiers, statistical significance values, and matched and complete metabolites.

### 4.6. Community Network Analysis

Metabolic processes are interdependent. The scope of this association may encompass causal, biological, and chemical connections [50]. In this study, we evaluated the degree of association between individual profiling metabolites and PK parameters or patient characteristics observed after single-dose ETO administration, utilizing the distance correlation metric as a measure of dependence [51]. We calculate the linear distance correlation (d) between variables, using Pearson’s correlation—to determine the sign or directionality of the distance correlation for each pair—but also a distance-based non-linear correlation with values between 0 and 1. Subsequent analyses were restricted to those comparisons exhibiting a d-value > 0.5 and an FDR p-value < 0.05.

A similarity matrix based on these correlations was used to construct a tripartite network in which metabolites (previously determined, selecting the differential variables by the limma method when comparing T0 and Tmax) were the links between PK parameters and patient characteristics (as listed in detail in the Table S1). Correlation values were used as weighted edges: metabolites, PK parameters, and patient characteristics as network nodes. Community structures within our graph were identified using the Leiden algorithm. The structure of the network and its constituent modules, or communities, were determined with the support of the igraph package [52].

## Figures and Tables

**Table 1:** Integrated pathways and community network analysis results.

Parameters	Molecule (ID HMDB/PubChem)	Biological/Chemical Class/Function/Pathway
Predictive T0 (Baseline metabolism pre-dose)		
Laboratory/Clinical characteristics		
Systolic pressure	dTDP-D-galactose (HMDB0006876)	Cholesterol biosynthesis = WP1795 Cholesterol synthesis disorders = WP5193.
	Mevalonic acid (HMDB0000227)	Cholesterol metabolism (WP5304)
Aspartate aminotransferase seric	L-Palmitoylcarnitine (HMDB0000222)	Carnitine metabolism (R-HSA-200425) An ester of carnitine that facilitates the transfer of fatty acids from the cytosol to the mitochondria during fatty acid oxidation.
Total bilurubin Direct bilirubin Indirect bilirrubin Leukocites urine	Dityrosine (HMDB0006045)	Dityrosine has also been found to be involved in oxidative/nitrative stress under a wide range of conditions and in a variety of biological systems.
Albumin seric Hemoglobin seric Erythrocytes seric Hematocrit seric	Palmitoleic acid (HMDB0003229)	Omega-9 fatty acid synthesis (WP4724)
Effect Tmax (Metabolism post-dose)		
Pharmacokinetic parameters		
AUC0-t (h·μg/mL) Cmax (μg/mL)	PS(PGF1alpha/20:5) (HMDB0282870)	Phospholipids with anti-inflammatory properties
	PS(PGF2alpha/18:1) (HMDB0281557)	Phospholipids with anti-inflammatory properties
Kel (1/h), T1/2 (h)	15-Epi-lipoxin A4 (HMDB0012587)	Tryptophan metabolism (WP465)
	Riboflavin (HMDB0000244)	Riboflavin Metabolism = map00740
	6-Ketoprostaglandin E1 (HMDB0004241)	Eicosanoid metabolism via cyclooxygenases (COX)
Laboratory/Clinicals characteristics		
Creatinine seric Fasting glucose seric Hemoglobin seric hematocrit seric Erythrocytes seric	L-Palmitoylcarnitine (HMDB0000222)	L-palmitoylcarnitine is an acylcarnitine. The acyl groups, organic acids and fatty acids are transported from the cytosol to the mitochondria for degradation to produce energy.
	Linoleic acid (HMDB0000673)	Belongs to the class of organic compounds known as linoleic acids and derivatives.
	Palmitoleic acid (HMDB0003229)	Peptide hormone metabolism = R-HSA-2980736, sSynthesis, secretion, and inactivation of Glucagon-like Peptide-1 (GLP-1) = R-HSA-381771/WP2728; free fatty acid receptors = R-HSA-444209, Omega-9 fatty acid synthesis = WP4724
	3-Oxooctadecanoic acid (HMDB0010736)	It belongs to the class of organic compounds called long-chain fatty acids.
Transaminase oxaloacetic acid (TGO) seric	Etodolac (HMDB0014887)	Drug
	Flavin mononucleotide (HMDB0001520)	Metabolism of nitric oxide: NOS3 activation and regulation (WP1850), riboflavin metabolism = map00740, cytosolic iron-sulfur cluster assembly = WP2690, metabolism of cofactors = WP4990, riboflavin and CoQ disorders = WP5037, vitamin B12 disorders = WP4271
Total bilirubin seric Indirect bilirubin seric Leukocytes seric Segmented neutrophils seric	Glucuronic acid (HMDB0000127)	Phosphatidylinositol phosphate metabolism = map00562
	Etodolac acyl glucuronide (HMDB0060916)	Metabolite drug
	Ascorbic acid (HMDB0000044)	Carnitine synthesis = R-HSA-71262/WP4996, catecholamine biosynthesis = R-HSA-209905, vitamin C (ascorbate) metabolism = R-HSA-196836, effects of nitric oxide = WP1995, metabolism of amine-derived hormones = WP3548, vitamin B12 metabolism = WP1533, pluripotent stem cell differentiation pathway = WP2848
Mean corpuscular volume	Vitamin A (HMDB0000305)	Metabolism of fat-soluble vitamins = R-HSA-6806667, signaling by retinoic acid = R-HSA-5362517, retinoid metabolism and transport = R-HSA-975634, triglyceride metabolism = R-HSA-8979227, digestion and absorption = R-HSA-8963743, vitamins A and D—action mechanisms = WP4342
Red cell distribution width	Arg-gly-asp-ser (HMDB0248573- PubChem 107775)	Tetrapeptide may play an important role in the cell attachment domain of fibronectin

The table lists the molecules that appear simultaneously in the results of pathway enrichment and network analysis. For each metabolite, the first column indicates the associated pharmacometric parameter and participant traits, either baseline or post-ETO. The last columns indicate the biological function and/or pathway defined by the enrichment analysis. Identificator Human Metabolome Database (HMDB), Reactome Homo Sapiens pathway (R-HSA), WikiPathways (WP), Public Chemical Databases (PubChem).

## Data Availability

The data presented in this study are available on request from the corresponding author.

## Supplementary Materials

The following supporting information can be downloaded at: https://www.mdpi.com/article/10.3390/ph18010082/s1, Table S1: Summary of Participant Characteristics. This table presents the descriptive statistics of demographic, clinical, and laboratory data of the participants included in the study; Table S2: Adverse events. Reported adverse events for some participants. Table S3: Pharmacokinetics parameters. Pharmacokinetics parameters were calculated for each participant using the concentration across time data; Table S4: Statistically differential compounds (pre-dose and post-dose) and their putative identifications; Obtained data for each compound from instrumental analysis. Putative annotation is added too; Table S5: Pathway enrichment results. List of enriched pathways and additional statistics and data; Table S6: Community results at pre-dose and post-dose. Results of community network analysis using relative abundance at pre-dose and post-dose of differential metabolites. Each module or community is composed of a metabolite linking to either a pharmacokinetic parameter or characteristic or both.
