# Supplementary material for: Etodolac Single Dose Metabolic Profile Elucidation: Pharmacokinetics and Adverse Events in Healthy Volunteers
Source: Pharmaceuticals (Basel). 2025 Jan 11;18(1):82. doi: 10.3390/ph18010082 (PMC11768370; doi:10.3390/ph18010082)
Supplement: Supplementary file 1 [file pharmaceuticals-18-00082-s001.zip › pharmaceuticals-3406101-supplementary.pdf]

Supplementary Files

1. Supplementary Methods

2. Supplementary Results

3. SupplementaryTables:

Table S1. Demographic, clinical, and laboratory characteristics. This table contains the descriptive statistics of demographic, clinical, and laboratory characteristics of the participants included in the study.

Table S2. Adverse events. Reported adverse events for some participants.

Table S3. Pharmacokinetics parameters. Pharmacokinetics parameters were calculated for each participant using the concentration across time data.

Table S4. Statistically differential compounds (pre-dose and post-dose) and their putative identifications

Table S5. Pathway enrichment results. List of enriched pathways and additional statistics and data.

Table S6. Community results at pre-dose and post-dose. Results of community network analysis using relative abundance at pre-dose and post-dose of differential metabolites. Each module or community is composed of a metabolite linking to either a pharmacokinetic parameter or characteristic or both.

1. Supplementary Methods

1. Volunteers and Study Design

The present study was nested in a single-dose, open-label, randomized pharmaco-bioequivalence study performed under biosafety and ethical standards. The reference formulation was an etodolac controlled-release coated tablet of 500 mg Flancox® with the administration of one single-dose tablet. The volunteers were hospitalized at 7:00 p.m. and had supper before 9:00 p.m. After an overnight fast, a specific high-calorie diet was administered 30 minutes before taking the medication. They received (at ~7:00 a.m.) a tablet of 500 mg of etodolac with 200 mL of water. Lunch: 4 hours after taking the medication. Afternoon snack: 8 hours after taking the medication. Dinner: 10 hours after taking the medication.

At 2 h before and 6, 8, and 12 h after the dose administration, vital signs were checked. The recruitment period was 24 h, during which period, blood samples (5 mL) from a suitable antecubital vein were collected, post-dosing via an indwelling catheter, into ethylenediaminetetraacetic acid (EDTA)-containing tubes at 0:15, 0:30, 0:45, 1:00, 1:15, 1:30, 1:45, 2:00, 2:15, 2:30, 2:45, 3:00, 3:20, 3:40, 4:00, 5:00, 6:00, 8:00, 10:00, 12:00, 24:00, and 36:00h. The blood samples were centrifuged at ~2,000×g for 10 min at 4°C, and the plasma was stored at -70°C until assayed. Samples were handled under yellow light due to the photosensitivity of the drug.

2. Determination of Pharmacokinetic Profile by LC-MS/MS

Chromatographic separations were performed in an LC-20AD analytical pump and SIL-20A HT autosampler from Shimadzu. The stationary phase employed an Agilent Polaris 5 µm C18-A 50 x

2,0mm column (Phenomenex). The mobile phase consisted of a solution of acetonitrile and 5 mM ammonium acetate, flowing at 0.3 mL/min in proportion (8:2). The autosampler was maintained at 22°C, with an injection volume of 5 µl and 2.5-minute duration. Mass spectrometry was conducted on a Quattro Micro mass spectrometer from Micromass, equipped with an electrospray ionization (ESI) source. While operating in negative ionization mode with nitrogen as the desolvation gas, ESI source parameters were set as follows: source temperature of 100°C, desolvation temperature of 450°C, desolvation flow of 800 L h^{-1}, and capillary voltage of 3,50 kV. Etodolac was detected by a multiple reaction monitoring (MRM) transition of 286 > 242 using a cone voltage of 30 eV, and mefenamic acid was detected by an MRM transition of 240 > 196 using a cone voltage of 30 eV. Data was acquired using MassLynx 4.1.

2.1. Preparation of Standards and Quality Controls

Etodolac stock solution (108.5 µg/mL) was prepared in methanol and diluted with H_2O to obtain the corrected stock solution (100 µg/mL) for the spiking calibration curve and quality controls (QC) samples. QC samples were prepared in the following levels: low, 0,6 µg/mL (LQC); medium, 50 µg/mL (MQC); high, 75 ug/mL (HQC); and a dilution, 42.5 µg/mL (DQC), calculated by taking the upper limit of quantification (ULOQ) of 100 µg/mL, adding 70%, and dividing by 4. Calibration curves in plasma were prepared to the final concentration range of 0,2, 0,4, 2, 5, 20, 40, 80, and 100 µg/mL. The internal standard was prepared in methanol.

2.2 Sample Extraction

Samples of plasma consisted of aliquots of 300 µL, and 25 µL of IS solution (orphenadrine) was added to the plasma, and 25 µL of HCl 1 M as well as 1,000 µL of a diethyl ether/dichloromethane (70:30 v/v) were added. Then, samples were shaken for 5 min and centrifuged at 12,879 g for 10 min at 4°C. Finally, the supernatant was collected and dried over a nitrogen gas (N_2) flow. An acetonitrile/water solution (80:20 v/v) was used for resuspension.

2.3. Method Validation

The analytical method was validated according to Agência Nacional de Vigilância Sanitária (ANVISA, RDC N° 27/2012), observing the following parameters: specificity, carryover, matrix effect, calibration curve, accuracy, precision, and stability. Specificity was ascertained by analyzing blank human plasma samples from six individuals and comparing the chromatograms among blank human plasma spiked with etodolac in the lower limit of quantification (LLOQ) concentration of 1 ng/mL and IS. The level of hemolysis used in the validation of this study was the highest level (very low, 1; low, 2; moderate, 3; high, 4). Carryover was ascertained by analyzing three injections of the same blank sample, one sample in the LLOQ concentration (1 ng/mL), and one sample in ULOQ (550 ng/mL) chromatograms in the following sequence: LLOQ sample, blank sample, ULOQ samples, and two blank samples. The matrix effect was ascertained by spiking eight different extracted blank human plasma samples (four normal, two lipemic, and two hemolyzed) with etodolac at QC concentrations and the IS. Peak areas of extracted spiked samples were compared to those of standard solutions.

The calibration curve was prepared using eight different blank human plasma samples, spiked with concentrations at eight levels ranging from 1 to 550 ng/mL. Analyte concentrations in samples were calculated by linear regression equation ($y = ax + b$) where y corresponds to the analyte/IS peak area ratio, and x corresponds to the ratio of etodolac to IS concentration. Due to the range of the calibration curve and the lower value of the sum of the relative errors of the nominal values of the calibration versus its values obtained by the curve equation, the weighting factor of reciprocal concentration squared ($1/x^2$) was applied. Intra- and inter-batch accuracy and precision were evaluated at five different levels (1, 3, 233.75, 275, and 412,5 ng/mL) of QC samples in quintuplicate in three different batches. The accuracy of the method was expressed as relative error (RE), whereas precision was obtained by calculating the within- and between-run coefficient of variation (CV). The acceptance

criteria for RE and CV must not exceed 15% for QC and 20% for LLOQ. All stability assays were performed to cover the conditions anticipated for handling real samples: freshly prepared, post-processing, short-term, freeze–thaw, and long-term were evaluated at concentrations of LQC and HQC. RE and CV were used to check possible variations.

2.4. Determination of Etodolac Pharmacokinetic

Following Food and Drug Administration (FDA) guidelines, blood samples were drawn up to a period of three to five times the terminal elimination half-life (t1/2), and the mean AUC0–t/AUC0–∞ ratio was required to be higher than 80%. The area under the concentration–time curve (AUC0–t) was calculated from time 0 to time t. The total area under the curve (AUC0–∞) was obtained up to the last measurable concentration, and extrapolations were performed using the last measurable concentration and the terminal elimination rate constant (Ke). The terminal elimination rate constant, Ke, was estimated from the slope of the terminal log10 transformed exponentially and multiplied by the −2.303 phase of the plasma of the etodolac concentration–time curve (by means of the linear regression method) adjusted in the last three values. The terminal elimination half-life, t1/2, was then obtained as 0.693/Ke. The C_{max} and the time to reach maximum plasma concentration (T_{max}) values were determined by visual inspection of the plasma etodolac concentration–time profiles. Results are presented as mean ± standard deviation (SD).

3. Metabolomics Analysis

An ACQUITY UPLC was used, coupled to a XEVO-G2XS Quadrupole Time-of-Flight (QToF) mass spectrometer (Waters, Manchester, UK) equipped with an ESI source, operated both in positive (+) and negative (-) ionization modes. Chromatographic analysis was performed using an ACQUITY UPLC® CSH C18 column (C18, 2.1 mm x 100 mm x 1.7 µm, Waters), employing the mobile phase A composed of water + 0.1% formic acid, and the mobile phase B, composed of acetonitrile + 0.1% formic acid. The flow rate was 0.4 mL min^{-1}. Initially, the column was conditioned with 10% B and maintained for 2 min, increasing to 40% B over the next 0.5 min, increasing to 90% B over the next 4.5 min, and staying for 2 min at that rate. In 2.0 min, mobile phase B returned to 10% and was maintained for another 2.0 min, equilibrating the column for the next injection. The total running time was 13 min. The injection volume was 1 µL (+/-). The mass spectrometer was operated in MS^E mode with an m/z range of 50–1200 Da and an acquisition time of 0.5s per scan. MS^E analysis was operated at 6 eV for low collision energy and a ramp of 20-50 eV for high collision energy. Leucine enkephalin (molecular weight = 555.62; 200 pg/mL in 1:1 ACN: H_2O, v/v) was used as the lockmass for mass accuracy, and a 0.5 mM sodium formate solution was used for calibration. Other parameters were as follows: source temperature = 140 °C, desolvation temperature = 550 °C, desolvation gas flow = 900 L/h, capillary voltage = 3.0 kV (+) / 2.8 kV (-), and cone voltage = 40 V. The LC-MS raw files were processed using the Progenesis™ QI software v2.4 (Nonlinear Dynamics, Newcastle, United Kingdom), which enabled the selection of possible adducts, peak alignment, deconvolution, and compound annotation based on MS^E experiments. The adducts $[M+H]^+$, $[M+K]^+$, $[M+Na]^+$, and $[M+H-H_2O]^+$ were considered for the positive acquisition mode, while $[M-H]^-$, $[M+Cl]^-$, $[M-H_2O-H]^-$, and $[M+FA-H]^-$ were considered for the negative acquisition mode. For each sample, Progenesis QI generates an intensity table of the ions, labeled according to their retention time and nominal masses, called features, as a function of their intensity (areas of the extracted ion chromatogram).

Due to low- and high-energy acquisition enabled by the use of MS^E, we have information on precursor ions (low energy) and fragments (high energy) in the same spectrum. Annotation of molecules considered mass accuracy (≤5 *ppm*), fragmentation profile (≤10 *ppm*), isotope similarity (>70%), and biological relevance. The in-house 'SDF2PQI' software was employed to enhance fragment match numbers for Progenesis PQI data compatibility with external SDF-based spectral libraries. The libraries used were MassBank of North America (MoNA), the Human Metabolome Database (HMDB), and the LIPID MAPS structure database.

2. Supplementary Results

Method Validation

The selectivity analysis for etodolac indicated that the interference peaks in all blank samples were below 1% compared to the lower limit of quantification (LLOQ) and IS. Chromatographic evidence showed no residual peaks in blank samples for either the analyte or the IS, confirming no carryover effect. The matrix effect for LQC and HQC samples was minimal at 4.74%, with no notable interference from matrix components across eight individual plasma samples.

Calibration curves demonstrated good linearity within the 1–550 ng/mL range, with individual regression equations and determination coefficients (r^2) as follows: (i) y= 0.00296424x + 0.000673827 (r^2 = 0.995983); (ii) y= 0.0034608x + 0.000175332 (r^2 = 0.997647); and (iii) y= 0.00296909x - 0.0000105154 (r^2 = 0.990637). QC concentrations showed relative error (RE) values within ±15%. Both intra- and inter-batch precision were under 10%, indicating consistent measurements across identical concentrations. Precision and accuracy for all assays were below 15%, showcasing stability throughout the testing period. Additionally, LQC and HQC were tested for drug stability in biological fluid. The conditions were: freshly prepared, post-processing (auto-injector, 73:43 hours), short-term (72:21 hours), freeze–thaw (4 cycles) at -20° C and -70°C and long-term stability at -20° C and -70°C for 90 days. All assays presented precision and accuracy values lower than 15%, demonstrating stability during the period tested.
